# Comparative and Phylogenetic Analysis of Complete Plastomes among Aristidoideae Species (Poaceae)

**DOI:** 10.3390/biology11010063

**Published:** 2022-01-02

**Authors:** Xiu-Xiu Guo, Xiao-Jian Qu, Xue-Jie Zhang, Shou-Jin Fan

**Affiliations:** Key Lab of Plant Stress Research, College of Life Science, Shandong Normal University, No. 88 Wenhuadong Road, Jinan 250014, China; 2018010081@stu.sdnu.edu.cn (X.-X.G.); quxiaojian@sdnu.edu.cn (X.-J.Q.)

**Keywords:** Aristidoideae, plastome, comparative genomics, phylogenomics, species diversification

## Abstract

**Simple Summary:**

Aristidoideae is a subfamily of Poaceae, including three genera, *Aristida*, *Stipagrostis*, and *Sartidia.* In this study, the plastomes of *Aristida adscensionis* and *Stipagrostis pennata* were newly sequenced, and a total of 16 Aristidoideae plastomes were compared. All plastomes were conservative in genome size, gene number, structure, and IR boundary. Repeat sequence analysis showed that forward and palindrome repeats were the most common repeat types. The number of SSRs ranged from 30 (*Sartidia isaloensis*) to 54 (*Aristida purpurea*). Codon usage analysis showed that plastome genes preferred to use codons ending with A/T. A total of 12 highly variable regions were screened, including four protein coding sequences and eight non-coding sequences. All Maximum Likelihood and Bayesian Inference trees strongly support the monophyly of Aristidoideae and each of the three genera. Within Aristidoideae, *Aristida* is sister to the clade composed of *Stipagrostis* and *Sartidia*. The divergence between C_4_ *Stipagrostis* and C_3_ *Sartidia* was estimated at 11.04 Ma, which may be associated with the drought event in the Miocene period. Finally, the differences in carbon fixation patterns, geographical distributions, and ploidy may be related to the difference of species numbers among these three genera. This study provides insights into the phylogeny and evolution of the subfamily Aristidoideae.

**Abstract:**

Aristidoideae is a subfamily in the PACMAD clade of family Poaceae, including three genera, *Aristida*, *Stipagrostis*, and *Sartidia.* In this study, the plastomes of *Aristida adscensionis* and *Stipagrostis pennata* were newly sequenced, and a total of 16 Aristidoideae plastomes were compared. All plastomes were conservative in genome size, gene number, structure, and IR boundary. Repeat sequence analysis showed that forward and palindrome repeats were the most common repeat types. The number of SSRs ranged from 30 (*Sartidia isaloensis*) to 54 (*Aristida purpurea*). Codon usage analysis showed that plastome genes preferred to use codons ending with A/T. A total of 12 highly variable regions were screened, including four protein coding sequences (*mat*K, *ndh*F, *inf*A, and *rpl*32) and eight non-coding sequences (*rpl*16-1-*rpl*16-2, *ccs*A-*ndh*D, *trn*Y-GUA-*trn*D-GUC, *ndh*F-*rpl*32, *pet*N-*trn*C-GCA, *trn*T-GGU-*trn*E-UUC, *trn*G-GCC-*trn*fM-CAU, and *rpl*32-*trn*L-UAG). Furthermore, the phylogenetic position of this subfamily and their intergeneric relationships need to be illuminated. All Maximum Likelihood and Bayesian Inference trees strongly support the monophyly of Aristidoideae and each of three genera, and the clade of Aristidoideae and Panicoideae was a sister to other subfamilies in the PACMAD clade. Within Aristidoideae, *Aristida* is a sister to the clade composed of *Stipagrostis* and *Sartidia*. The divergence between C_4_ *Stipagrostis* and C_3_ *Sartidia* was estimated at 11.04 Ma, which may be associated with the drought event in the Miocene period. Finally, the differences in carbon fixation patterns, geographical distributions, and ploidy may be related to the difference of species numbers among these three genera. This study provides insights into the phylogeny and evolution of the subfamily Aristidoideae.

## 1. Introduction

The subfamily Aristidoideae, together with Panicoideae, Chloridoideae, Micrairoideae, Arundinoideae, and Danthonioideae, forms the PACMAD clade of Poaceae [1]. The most striking feature of Aristidoideae is that they contain three awns at the top of their lemma. The inflorescence type of Aristidoideae has spread or contracted panicles, with only one fertile floret in one spikelet. Their leaves are narrow and usually rolled longitudinally, which is related to their adaptation to arid environments [2,3]. Three genera, *Aristida*, *Stipagrostis*, and *Sartidia*, are contained in Aristidoideae. The core genus is *Aristida*, with about 300 species, which are widely distributed in temperate and subtropical arid areas [4]. There are about 50 species in *Stipagrostis*, which distributed in deserts and semi-deserts [5,6]. Only six species are contained in *Sartidia*, and they often occur in grasslands and savannas [3,6]. In addition, research on this subfamily has focused on the origin of C_3_ and C_4_ [5,7,8,9]. Aristidoideae has twice-independent C_4_ origins, once in *Aristida* and the other in *Stipagrostis*. *Aristida* (except for *A. longifolia*, which is the earliest diverging taxa in *Aristida* and it’s a C_3_ plant) and *Stipagrostis* are C_4_ photosynthesis species, while *Sartidia* species are C_3_ plants [5].

The subfamily Aristidoideae is monophyletic, and each of the three genera in the subfamily is also monophyletic [10]. However, the relationship of Aristidoideae with other subfamilies in the PACMAD clade remains in question. In the molecular phylogenetic tree of Poaceae, updated by Grass Phylogeny Working Group (GPWG) in 2012, the subfamily Aristidoideae is the basal group of the PACMAD clade [1]. The phylogenetic study of the PACMAD clade, based on the chloroplast genome data, indicated that the subfamily Panicoideae is the basal group of this clade and Aristidoideae formed a sister group relationship with the rest of subfamilies, however, only one Aristidoideae species was included [11]. The phylogenetic study by Saarela, et al. [12] sampled more species in Aristidoideae, but the position of the Aristidoideae in PACMAD remains controversial. Two sets of chloroplast genome data support Panicoideae as being the basal group of PACMAD, and three sets of chloroplast genome data support that Aristidoideae is the basal group of PACMAD; the complete chloroplast genome coding region, excluding gapped sites but containing positively selected sites, approve that Panicoideae and Aristidoideae are sister groups. While the data based on the mitochondrial gene *mat*R and seven highly variable mitochondrial gene intron sequences (*cox*2 intron, *nad*1 intron 2, *nad*4 intron 1, and *nad*7 introns 1, 2, 3, and 4.) support that Aristidoideae and Panicoideae are sister groups to each other and form the base group of PACMAD together [11].

The phylogenetic relationships of these three genera in Aristidoideae have always been controversial in grass taxonomy. The sister relationship of *Aristida* and *Stipagrostis* is supported by both six chloroplast gene fragments and the nuclear ppc multigene family [7,13]. However, neither study sampled *Sartidia* species, due to the low species number and narrow distribution of this genus. The combined analysis, based on chloroplast *trn*L-F, *rpl*16 and nuclear ITS sequences, support *Aristida* to be the sister to the clade composed of *Stipagrostis* and *Sartidia* [10]. The same conclusion was made by the Grass Phylogeny Working Group II [1], based on three chloroplast markers (*rbc*L, *ndh*F, *trn*K/*mat*K). However, both studies included only one *Sartidia* species. Morphologically, the spikelets of *Aristida* are more similar to those of *Sartidia*, and there are no feathery hairs on their awns [10]. The embryo proportion (the ratio of embryo length to caryopsis length) of these two genera is 1/5–2/5, while it is 1/3–1/2 for *Stipagrostis* [14,15]. However, the caryopsis of *Aristida* and *Stipagrostis* are cylindrical, which have been laterally compressed, and the caryopsis of *Sartidia* is dorsally compressed [2,14,16]. In the anatomical structure, there are two layers of vascular bundle sheath cells in all Aristidoideae species. Only the outer layer of vascular bundle sheath are parenchyma cells in *Sartidia* and *Stipagrostis*, while in *Aristida*, two layers of vascular bundle sheath are parenchyma cells [3,5]. Therefore, neither molecular nor morphological evidence could explain the relationship between the three genera well. In this study, the whole plastomes were used to reconstruct the relationships among three genera of Aristidoideae. Representative species of three genera were sampled to test the phylogenetic resolution ability of plastome.

The chloroplast is a structure where green plants carry out photosynthesis and carbon fixation. It is a semi-autonomous organelle, which is ubiquitous in land plants, algae and some protists [17,18]. The first published plant plastome sequences were *Nicotiana tabacum* L. [19] and *Marchantia polymorpha* L. [20]. The plastome of angiosperms is a double-stranded ring with a highly conserved quadripartite structure: a large single-copy region (LSC), a small single-copy region (SSC), and two inverted repeat (IR) regions. The IR regions are the areas that expand or contract during the evolution of the plastome, which is also the main reason for the difference in the size of the plastomes [21]. The plastome usually contains 101–118 different genes [22], and the genome size ranges from 120 kb to 160 kb [17], but there are some exceptions [23,24,25]. The largest known angiosperm plastome is *Pelargonium* × *hortorum*, with a length of 217,942 bp [26]. The plastome is moderate in size and contains considerable genetic information. It has a good collinearity with plastome of various plant groups, and the nucleic acid replacement rate in chloroplast DNA is sufficient [27]. In addition, the significant difference of molecular evolution speed between the coding and non-coding regions of the plastome made it suitable for the phylogenetic study of different taxonomic levels [28]. Phylogenetic genomics based on plastomes has developed rapidly in recent years [29,30,31].

In the current study, the plastomes of two newly sequenced Aristidoideae species were reported. The aims of this study were to: (1) compare the plastome structure of the 16 Aristidoideae species; (2) screen the highly variable markers for Aristidoideae, especially within the genus *Aristida*; (3) explore the phylogenetic position of Aristidoideae, as well as the intergeneric relationships of *Aristida*, *Stipagrostis*, and *Sartidia*, and interspecific relationships of *Aristida*. All in all, this is the first comparative study in Aristidoideae based on plastid genomes data. The application of these results will contribute to the evaluation of phylogenetic relationships and biogeographical studies among close relatives of Aristidoideae.

## 2. Materials and Methods

### 2.1. Plant Material, DNA Extraction, and Sequencing

The plants of *Aristida adscensionis* Linnaeus and *Stipagrostis pennata* (Trinius) De Winter were collected from Taohuayu in Shandong Province, Chinaand Bu’erjin in Xinjiang Uygur Autonomous Region, China. The voucher specimens were deposited in the herbarium of Shandong Normal University (SDNU), where the voucher specimen of *A. adscensionis* was registered under the number 20061013-1, and the voucher specimen of *St. pennata* was registered under the number 608068. Total genomic DNA were isolated from silica-dried leaves using a modified CTAB method [32]. Agarose gel electrophoresis was used to detect the quality of extracted DNA. A NanoDrop 2000 spectrophotometer (Thermo Scientific, Wilmington, DE, USA) was used to determine the concentration of the isolated DNA.

A total of 0.2 μg DNA was used for the DNA library preparations. The sequencing library was generated using NEB Next^®^ Ultra™ DNA Library Prep Kit for Illumina (NEB, Beijing, China) according to the manufacturer’s recommended instructions. The genomic DNA was broken into a size of ~350 bp. The resulting fragments were endpolished, A-tailed, and ligated with the full-length adapter for Illumina sequencing, followed by further PCR amplification. The 150 bp paired-end sequencing was performed with the Illumina NovaSeq 6000 platform in Novogene (Beijing, China).

### 2.2. Genome Assembly and Annotation

The plastomes of *Aristida adscensionis* and *Stipagrostis pennata* were assembled with Organelle Genome Assembler (OGA, https://github.com/quxiaojian/OGA (accessed on 6 February 2021)) [33]. The sequences of all 16 species were annotated with Plastid Genome Annotator (PGA, https://github.com/quxiaojian/PGA (accessed on 14 February 2021)) [34] and manually corrected in Geneious v8.0.2 (https://www.geneious.com (accessed on 9 March 2021)). OrganellarGenomeDRAW (OGDRAW) v1.3.1 (https://chlorobox.mpimp-golm.mpg.de/OGDraw.html (accessed on 28 May 2021)) [35] was used to draw the plastome map. The plastomes information, such as the number of genes, gene length, GC content, and intron number, etc., was counted by Geneious v8.0.2. The sequence data have been submitted to the GenBank database under accession number MZ373986 and MZ375985.

### 2.3. Repeat Sequences and SSR Analysis

Repeat sequence analysis, including forward, reverse, complement, and palindrome repeats, was carried out by the REPuter website (https://bibiserv.cebitec.unibielefeld.de/reputer/ (accessed on 13 September 2021)) [36]. The parameters used in the analysis were as follows: the hamming distance was three, the maximum computed repeats was 50 bp, and the minimal repeat size was 30 bp. Simple sequence repeats (SSR) or microsatellites in the plastomes were detected by Perl script MISA [37]. The repeat units were set to 10, 5, 4, 3, 3, and 3 for mono-, di-, tri-, tetra-, penta-, and hexa-nucleotides, respectively [38]. The correlation analysis between SSR and chloroplast genome length and GC content were completed by the software SPSS v18.0 (SPSS Inc., Chicago, IL, USA).

### 2.4. Codon Usage Analysis

The codon usage of 16 Aristidoideae species was compared. The assessments of codon usage preference included the relative usage of synonymous codons (RSCU) and the effective number of codons (ENC). If the RSCU value is greater than 1, it means that the codon is used more frequently. ENC can be used to measure the degree of codon preference. The lower the value is, the stronger the codon preference of the gene is [39,40]. CodonW v1.4.2 (http://sourceforge.net/projects/codonw/ (accessed on 1 June 2021)) was used for codon usage analysis. The aligned coding sequences (CDSs) longer than 300 bp were picked, to ensure accuracy [41].

### 2.5. Comparative Genome Analysis and Divergent Hotspot Regions

The conversion of GenBank annotation files to mVISTA format files was completed by Perl script (https://github.com/quxiaojian/Bioinformatic_Scripts/get_mVISTA_format_from_GenBank_annotation.pl (accessed on 31 May 2021)). Plastome sequence alignment was performed by the online genome alignment tool mVISTA (http://genome.lbl.gov/vista/index.shtml (accessed on 4 June 2021)), and the alignment program used was Shuffle-LAGAN [42]. *St. pennata* was selected as the reference. MEGA v7.0.26 was used for DNA sequence polymorphism analysis [43]. The percentage of parsimonious information sites (Pi%) of CDS and non-coding regions were calculated for those with sequence length greater than 200 bp [44].

### 2.6. Phylogenetic Analysis and Taxon Removal Test

To avoid data duplication, one IR region was deleted before phylogenetic analysis. Based on plastome data, the phylogenetic relationship of the PACMAD clade and the subfamily Aristidoideae were reconstructed, respectively. A total of 22 plastomes were used to study the phylogeny of the PACMAD clade with three species (*Brachyelytrum aristosum*, *Bambusa bambos*, and *Oryza sativa*) as the outgroup. About two or three representatives of each subfamily in PACMAD clade were selected, however, six Aristidoideae representatives were selected, to reconstruct the phylogenetic relationship of PACMAD. For reconstruction of the phylogeny of Aristidoideae, a total of 19 plastomes were used, including ten *Aristida* species, three *Sartidia* species, three *Stipagrostis* species, and three species as an outgroup (*Sorghum bicolor*, *Zea mays*, and *Setaria viridis*). The species and their accession numbers used for phylogenetic analysis were shown in Table 1. Three data matrices (complete plastome, CDS, and IGS) were selected for phylogenetic analysis. The sequence alignment was performed by MAFFT v7.313 with default parameters [45]. The software jModelTest v2.1.6 was used to select the best nucleotide substitution model, according to the Akaike Information Criterion [46,47]. Maximum Likelihood analyses were conducted by RAxML v 8.0.26 [48], with the substitution model of GTRGAMMAI and 1000 bootstrap replicates.

The software MrBayes v3.2.7 was used to reconstruct the Bayesian Inference (BI) tree [49]. The Markov Chain Monte Carlo (MCMC) was run for 1,000,000 steps with a random starting tree, birth–death default priors, and we sampled one tree every 1000 steps. The birth–death model has given a framework for studying the rate of species formation, extinction and net diversification. Finally, we referred to much of the literature about Bayesian tree inference of Poaceae based on chloroplast genome sequences, and “birth–death” prior is frequently used in these literatures. The first 25% of steps were discarded as burn-in. The molecular dating analysis was conducted by treePL and TreeAnnotator v1.8.945, with the relaxed molecular clock [50,51]. Rapid relaxed clock dating is frequently applied to analyze large data sets with hundreds of sequences in phylogenomics, due to its accuracy and efficiency. The relaxed molecular clock method can accommodate the changes of molecular evolutionary rate between lineages over time. The minimum and maximum age for the crown of Aristidoideae and Panicoideae was set to 36.9 and 51.9 Ma, respectively, based on previously analyzed results [52]. The minimum and maximum age for the crown of Aristidoideae was set to 10 and 20.4 Ma, respectively, based on results of a previous study [53].

## 3. Results

### 3.1. Plastome Characteristics of Aristidoideae

**Figure 1:**
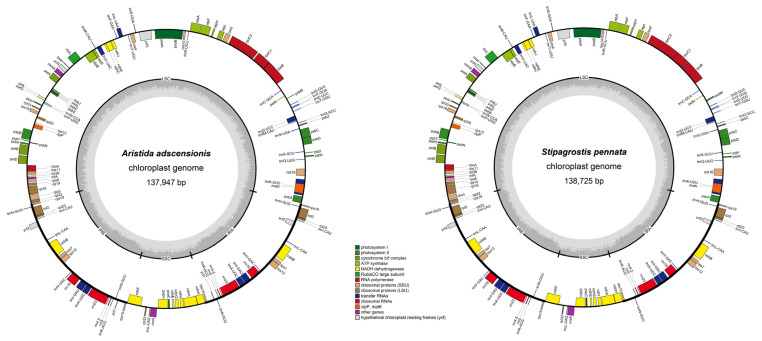
Maps of the newly sequenced plastome of *Aristida adscensionis* and *Stipagrostis pennata*. Different color blocks on the outer ring represent genes with different functions. The genes outside the outer ring were transcribed in a counterclockwise direction, while the genes inside the outer ring were transcribed in a clockwise direction. The gray dotted area in the inner circle represents the GC content of the plastomes.

The structural characteristics of 16 Aristidoideae plastomes were analyzed. All 16 plastomes showed a typical quadripartite structure, including a large single-copy region (LSC, 79,421–80,927 bp), a small single-copy region (SSC, 12,348–12,592 bp), and two inverted repeat regions (IR, 19,963–22,797 bp). The total length of these plastomes ranged from 132,603 (*Aristida* *glaziovii*) to 138,725 bp (*Stipagrostis pennata*), with a GC content of about 38.5% (ranging from 38.3% to 38.6% with the average of 38.44%). The IR regions had the highest GC content of 43.9–44.3%, followed by LSC (36.2–36.4%) and SSC (32.3–32.9%). Furthermore, all ten *Aristida* plastomes encoded 134 functional genes, with 88 protein-coding genes, 38 tRNA genes, and 8 rRNA genes. Three *Stipagrostis* species and three *Sartidia* species encoded 132 functional genes, including 86 protein-coding genes, 38 tRNA genes, and 8 rRNA genes (Table 2, Figure 1).

The gene order in Aristidoideae plastomes were basically the same, and no gene rearrangement, such as inversion events, were detected. It was also found that the *acc*D gene had been completely degraded in the whole subfamily. For the *ycf*1 gene, there were only 120 bp fragments found in *Aristida*. The *ycf*2 gene had residues of different lengths in these three genera, and the sizes of the remained fragments ranged from 105 bp to 792 bp. In Aristidoideae species, the *ycf*3 and *rps*12 genes contained two introns, and a total of eight genes (*ndh*B, *ndh*A, *rpl*2, *rpl*16, *pet*B, *atp*F, *pet*D, and *rps*16) included one intron. The *clp*P gene had lost two introns, while the *rpo*C1 gene had lost one intron (Figure 1).

### 3.2. Repeat Sequence Analysis

**Figure 2:**
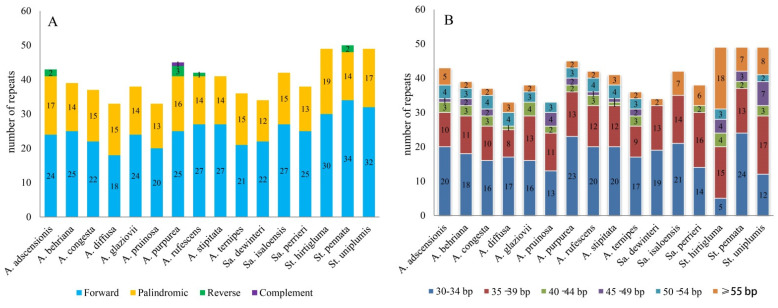
Repeat sequence analysis of plastomes in 16 Aristidoideae species. (**A**) The number of four types of repeats in different species. (**B**) The number of repeats with different lengths in different species.

Repeat sequences included interspersed repeat sequences and simple sequence repeats (SSR). A total of 649 (33–50 of each species) interspersed repeat sequences including 403 forward repeats, 237 palindromic repeats, eight reverse repeats, and one complement repeat were identified for 16 Aristidoideae plastomes. Forward repeats (18–34 of each species) and palindrome repeats (12–19 of each species) were found in all species. While reverse repeats were only detected in *A. adscensionis*, *A. purpurea*, *A. rufescens*, and *St. pennata*, and complementary repeat was only detected in *A. purpurea* (Figure 2A). In Aristidoideae, most of the repeat units were composed of 30–34 bp (42.373%) and 35–39 bp (30.354%), followed by repeat units > 55 bp (10.940%), 50–54 bp (6.163%), 40–44 bp (5.547%), and 45–49 bp (4.468%) (Figure 2B).

There were 708 simple sequence repeats in Aristidoideae plastomes, including 415 mononucleotide repeats, 130 dinucleotide repeats, 42 trinucleotide repeats, 111 tetranucleotide repeats, nine pentanucleotide repeats, and one hexanucleotide repeat (Table S1). The majority of mononucleotide SSRs were composed of A/T, only one repeat of “C” was detected in *Sa. isaloensis*, and one repeat of “G” was detected in *St. pennata*. Three types of dinucleotide repeats AT/TA/TC were found in Aristidoideae, the AC repeat appeared in *A. purpurea* only once. In addition, there were four types of trinucleotide repeats (AAT/AGA/TTC/CAT), eighteen types of tetranucleotide repeats (AAAT/AACG/AATA/AGAA/ATAG/ATCC/ATTT/CTTT/GAAA/GTAG/TAAA/TATC/TATT/TCGT/TTAT/TTCG/TTCT/TTTA) and seven types of pentanucleotide repeats (AATAG/ATAGA/ATTAG/TATTT/TCTAT/TTAGA/TTTTA). SSRs are more abundant in LSC than in SSC and IR. The vast majority of SSR, all compound SSRs, and pentanucleotide repeats are distributed in the LSC region (Table S1). The correlation analysis between various types of SSRs and chloroplast genome size and GC content showed that the plastome size was significantly negatively correlated with the proportion of single nucleotide repeats, and significantly positively correlated with the proportion of dinucleotide repeats. The GC content of the plastome was significantly positively correlated with the proportion of trinucleotide repeats (Table 3). The statistical SSR location information is listed in Tables S2 and S3.

### 3.3. Codon Usage Analysis

**Figure 3:**
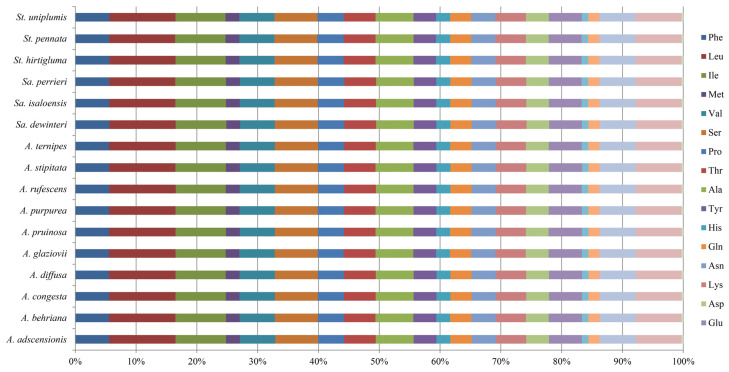
Comparison of the amino acid composition in the plastomes of 16 Aristidoideae species.

A total of 50 CDSs were selected for codon preference analysis (Figure 3). The number of codons ranged from 16,986 (*A. adscensionis*) to 17,101 (*St. hirtigluma* and *St. uniplumis*). The number of effective number of codons (ENC) ranged from 49.55 to 49.89 (Table 4). The Aristidoideae plastomes preferred to use synonymous codons ending with A (0.4226–0.4263 for each species) or T (0.4613–0.4643 for each species), while the content of G + G (GC3s) in the third synonymous codon was 0.268–0.273 for each species. The relative synonymous codon usage (RSCU) of all species were 0.28 (CUG) to 2.04 (UUA). Except for Met and Trp, which were encoded by only one codon, with the RSCU = 1, the RCSU values showed that UCA (S) (0.99–1.01 of each species) had almost no preference. Leucine (10.859–10.944%) was the most abundant amino acid for Aristidoideae plastomes, while cysteine (1.052–1.078%) was the least, except in stop codons (0.292–0.294%). There were no significant differences in codon content and frequency of optimal codons among Aristidoideae, and the codon adaptation index (CAI) ranged from 0.166 to 0.168 (Table S4).

### 3.4. Expansion and Contraction of the IR Region

**Figure 4:**
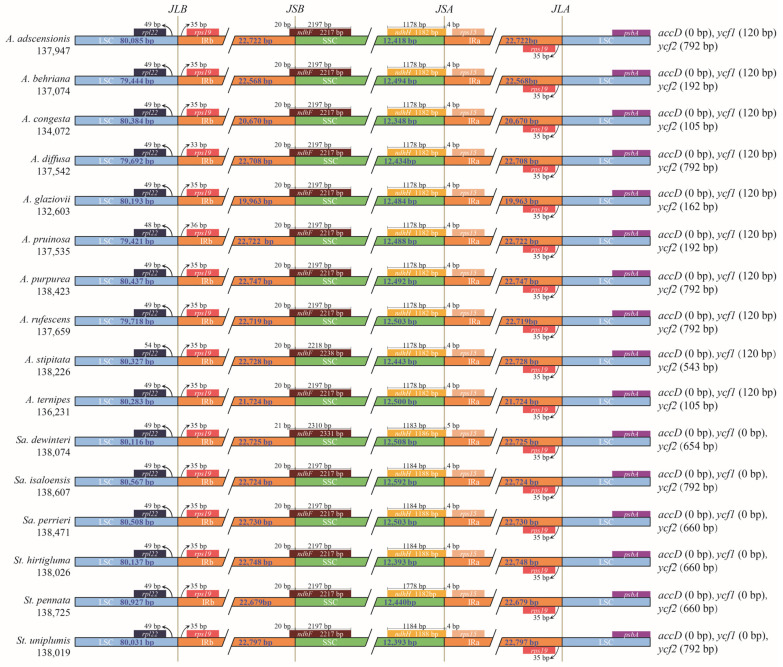
Comparison of boundaries of LSC, SSC, and IRs among 16 species in Aristidoideae. JLB: LSC/IRb junctions; JSB: SSC/ IRb junctions; JSA: SSC/ IRa junctions; JLA: LSC/IRa junctions. The gene names behind each species represents the lost genes in this species, and the number in parentheses represents the length of the residual gene fragment.

The boundaries of IR/SC for 16 Aristidoideae species were comprehensively compared (Figure 4). The LSC/IRb junctions (JLB) of all Aristidoideae were between *rpl*22 and *rps*19. The length of *rpl*22-*rps*19 in LSC was 49 bp for nearly all species, except that it was 33 bp in *A. diffusa*, 48 bp in *A. pruinosa*, and 54 bp in *A. stipitata*. The *rpl*22-*rps*19 length in IRb was 35 bp, with one exception that the length in *A. pruinosa* was 36 bp. The SSC/IRb junctions (JSB) of Aristidoideae were located in *ndh*F, and there were 20 to 21 bp of *ndh*F duplicated in IRb. The gene *ndh*H spanned the SSC/IRa junctions (JSA) for Aristidoideae, and only 4–5 bp in the 5′ end of *ndh*H existed in IRa region. The IRa/LSC junctions (JLA) were located in the intergenic region *rps*19-*psb*A. The length of *rps*19-*psb*A located in the IRa region was 35 bp for most taxa, but 36 bp in *A. pruinosa*.

### 3.5. Comparative Genome Analysis and Identification of Hypervariable Regions

**Figure 5:**
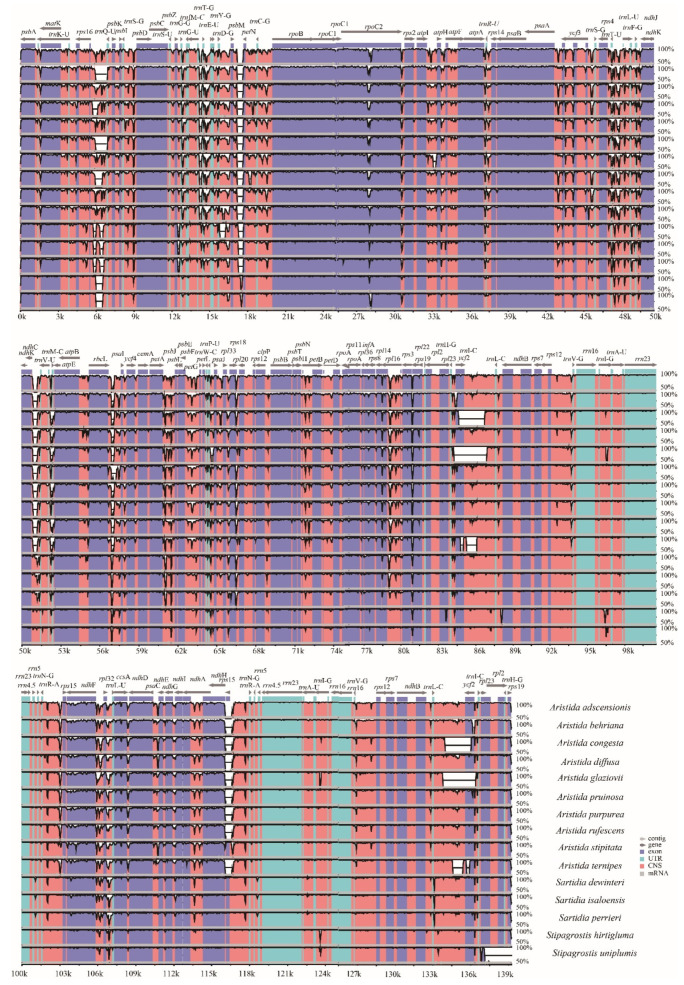
The homologous comparison of 16 Aristidoideae plastomes by mVISTA, with *St. pennata* as a reference. The horizontal axis, which represents the percentage of identity, ranges from 50–100%. Different colors indicate different gene regions.

With reference to *St. pennata*, the structural differences among Aristidoideae plastomes were compared by mVISTA (Figure 5). The *Aristida* species had a similar structure, while the structures of *Sartidia* and *Stipagrostis* plastomes were more similar. For the four parts of the plastome, the SC region had a greater degree of variation than the IR region, and most variation occurred in the non-coding region.

**Figure 6:**
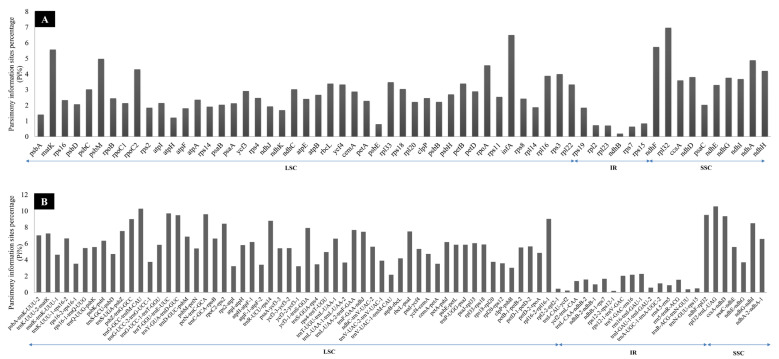
Comparison of the percentage of parsim-info sites in 16 Aristidoideae plastomes. (**A**) Protein coding sequences (CDS). (**B**) The introns and intergenic spacers (IGS).

To compare the sequence divergence of Aristidoideae plastomes, the parsimonious information sites were counted for CDS and non-coding sequences. The percentage of parsim-info (Pi%) sites for CDS ranged from 0.196 (*ndh*B) to 6.965 (*rpl*32), with an average value of 2.823 (Figure 6A). For non-coding sequences, the Pi% value varied from 0.185 (*rps*12 intron) to 10.563 (*rpl*32-*trn*L-UAG), and the mean value was 4.694 (Figure 6B). In the CDS, with Pi% ≥ 5 as the threshold value, four highly variable sequences (*mat*K, *ndh*F, *inf*A, and *rpl*32) were screened. Similarly, with the criterion of Pi% ≥ 9, there were eight highly variable sequences (*rpl*16 intron, *ccs*A-*ndh*D, *trn*Y-GUA-*trn*D-GUC, *ndh*F-*rpl*32, *pet*N-*trn*C-GCA, *trn*T-GGU-*trn*E-UUC, *trn*G-GCC-*trn*fM-CAU, and *rpl*32-*trn*L-UAG) that were filtered among non-coding sequences.

### 3.6. Phylogenetic Analysis and Molecular Dating

**Figure 7:**
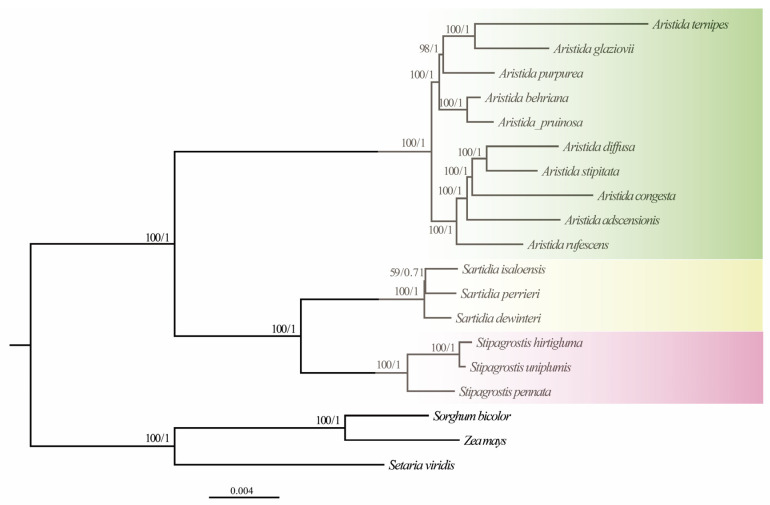
The Maximum Likelihood and Bayesian Inference trees of 16 Aristidoideae species based on their complete plastomes. The obtained bootstrap values (BS) and Bayesian Inference posterior probabilities (PP) are marked above the tree node (BS/PP).

**Figure 8:**
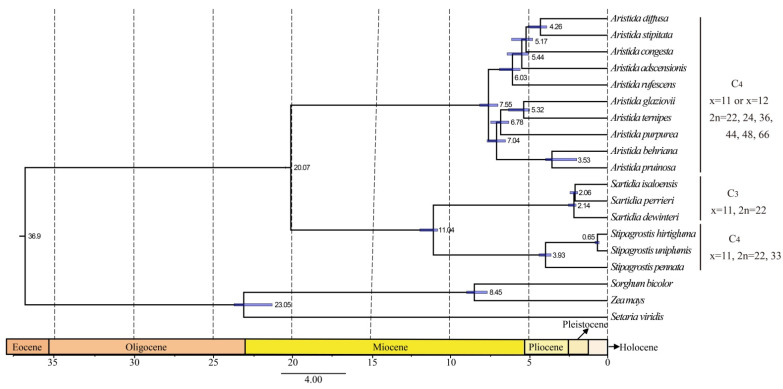
Chronogram constructed from treePL divergence estimation analysis. The short blue bar represents 95% confidence interval.

To determine the placement of Aristidoideae, the phylogenetic relationship of PACMAD was reconstructed based on the complete plastomes. The Maximum Likelihood tree supports Aristidoideae to be a sister to Panicoideae, with a bootstrap value of 100 (Figure S1). The clade composed of Aristidoideae and Panicoideae is firstly diverged among the PACMAD clade. In the present study, the phylogenetic position of Aristidoideae, based on three data sets, were reconstructed with three Panicoideae species as outgroups (Figure 7 and Figures S2–S5). The Maximum Likelihood and Bayesian Inference trees constructed from different data sets showed similar topologies. There is no doubt that Aristidoideae is a monophyletic group with good support (BS = 100, PP = 1). All data strongly supported that *Aristida*, *Stipagrostis*, and *Sartidia* were to be recognized as monophyletic groups (BS = 100, PP = 1). It is well supported that *Sartidia* and *Stipagrostis* are sisters to each other, and then form a sister relationship with *Aristida* (BS = 100, PP = 1). *Aristida* formed two clades, *A. behriana*, *A. pruinosa*, *A. purpurea*, *A. ternipes*, and *A. glaziovii* formed a monophyly, and they were sister groups with the monophyly formed by *A. adscensionis*, *A. congesta*, *A. diffusa*, *A. stipitata*, and *A. rufescens* (BS = 100, PP = 1). The estimated divergence time between *Stipagrostis* and *Sartidia* is at 11.04 Ma (HDP 95% = 10.47–12.18 Ma) in the Miocene period (Figure 8). The estimated divergence time of *Sartidia* is at 2.14 Ma (HDP 95% = 1.87–2.72 Ma) in the Pleistocene period.

## 4. Discussion

### 4.1. Basic Information of the Aristidoideae Plastomes

The size and structure of plastomes in most higher plants are relatively conservative [54]. The plastome size of angiosperms is generally 120–160 kb, and the length of the plastomes in Aristidoideae species is 132–138 kb, which is consistent with the length characteristics of plastomes in angiosperms [26]. There are 1–6 kb differences in length among the plastomes of 16 Aristidoideae species, which are caused by gene losses and length variation of intergenic regions. The genes *ycf*1 and *ycf*2 are the two longest genes in Aristidoideae plastomes. The full name of these two ycf genes is hypothetic chloroplast open reading frame. The function of protein YCF encoded by *ycf*1 or *ycf*2 is unknown, but some studies have shown that protein YCF is very important for plant survival [55]. In this study, the gene *ycf*1 is completely lost from *Sartidia* and *Stipagrostis* species, while there are only 120 bp fragments in *Aristida* species. The retained nucleotides of *ycf*2 varied from 105 to 792 bp for the 16 Aristidoideae species. The loss of *ycf*1 and *ycf*2 is similar to previously reported chloroplast genomes of Poaceae [30,56,57]. The genes *ycf*1/2 have a higher degree of variation than the commonly used molecular maker *mat*K and are suitable for phylogenetic research [58]. The gene *acc*D encodes the carboxyltransferase *β* subunit of acetyl-coenzyme A carboxylase, which is the rate-limiting enzyme for lipid synthesis [59]. The *acc*D gene has been completely lost in all 16 Aristidoideae species, and this is very common in Poaceae [60]. In this study, no changes in the order and direction of the chloroplast genes were detected.

Repetitive sequences play a crucial role in the structural rearrangement of plastomes [61]. Studies have shown that repeats were necessary for indels and replacement [62]. The existence and abundance of repeats in chloroplasts or the nuclear genome may be related to a variety of phylogenetic signals [63,64,65]. In this study, the different abundances and types of repeats in Aristidoideae species may provide additional evolutionary information. Among the 16 species of Aristidoideae, the number of repeats varied from 33 to 50, which is similar to the number of repeats previously reported in Poaceae [30,56]. *St. pennata* has the largest number of repeats, and its chloroplast genome size is the longest. *A. diffura* and *A. pruinosa* have the lowest number of repeats, but their chloroplast genome size is not the shortest, which is inconsistent with the previously reported rule that the larger the genome length is, the more repeats there are [66]. In terms of repeat types, the forward repeats were the most, followed by palindromic repeats and reverse repeats, which were consistent with the results of previous studies regarding Eragrostideae and *Gentiana* [56,67]. Forward repeats are often related to the activity of transposons, and the activity of transposons will lead to changes in gene structure. Forward repeats are usually used as markers for population genetic research [68]. Complementary repeats were the least common repeat types, and were not found in the chloroplast genomes of Eragrostideae [56], *Cleistogenes* [69], or *Avena* [30]. Among the 16 Aristidoideae species analyzed in this study, only one complementary repeat was detected in *A. purpurea*. SSRs are simple sequence repeats, which are widely distributed in the plastome. Due to a high variation degree, SSRs can be used as molecular markers for phylogenetic inference, population genetics, and biogeography [62,70,71]. A total of 712 SSRs were identified in Aristidoideae, with an average of 44.5 SSRs per species, and more than half were single nucleotide repeats. A total of 81.7% SSRs were located in the LSC region, which was close to previously reported plastomes of *Avena*, *Gentiana*, and *Pterocarpus* [30,67,72]. Correlation analysis showed that there was no correlation between the plastome size and the number of total SSRs in Aristidoideae species, which was consistent with the previous results in *Symplocarpus* [73]. However, there is a significant positive correlation between the plastome size and the proportion of dinucleotide repeats, which may greatly contribute to plastome size. GC content was significantly positively correlated with the proportion of trinucleotide repeats, including these four types of repeat units: AAT, AGA, CTA, and TTC, in 16 Aristidoideae species. SSRs distribution information showed that single nucleotide repeats could provide more parsimony information sites, while polynucleotide repeats were more conservative.

Codons are degenerate. Except methionine and tryptophan, other amino acids are encoded by 2–6 synonymous codons. However, the frequency of synonymous codon usage was different among plastomes [74]. It was generally believed that synonymous codon usage was not random and is species-specific. The analysis of codon preference would provide useful information for understanding species adaptability and molecular evolution [75]. Codon usage preference is influenced by many factors, such as GC content, gene length, tRNA abundance, mutation preference, and gene expression level [76,77,78]. This study revealed that chloroplast genes in Aristidoideae species preferred to use codons ending with A/T, which is consistent with previous studies in other groups [79,80,81,82]. ENC is an important index used to measure codon preference. If the value of ENC is less than 35, the codon bias are strong, and vice versa [83]. The ENC values of plastome genes among Aristidoideae species ranged from 49.55 to 49.85, which indicated that there was a weak codon usage preference in Aristidoideae species. All analyzed parameters suggested that the codon usage of Aristidoideae was relatively conservative, which was in line with the previous research results in Poaceae [56,80].

There are four boundaries between IR and SC regions of the plastome, IRb/LSC, IRb/SSC, IRa/LSC and IRa/SSC. The variation of the chloroplast genome size is frequently caused by the contraction and expansion of IR regions [84,85]. However, in Aristidoideae, the IR boundaries were very conservative, which were consistent with plastomes of Poaceae taxa [41,86]. The gene *rps*19 was located in the IRb region for Aristidoideae, while it was located in the LSC for *Amborella* [87], and the same expansion of IRb was found in *Eragrostis*, *Cleistogenes*, and *Miscanthus* of Poaceae [56,69,88]. About 20 bp of *ndh*F extend to the IRb region in the PACMAD clade of Poaceae [86], which is consistent with the observed structure of JSB for Aristidoideae. Almost all the nucleotides of *ndh*H located in IRa migrate into SSC region in the PACMAD clade [86]. For Aristidoideae, only 4–5 bp in the 5′ end of *ndh*H still remain in the IRa region.

### 4.2. Phylogenetically Informative Markers

It was noted that the Pi% value of the non-coding region (the mean Pi% = 4.694) was significantly higher than that of the coding region (the mean Pi% = 2.823), which was similar to previous studies [28,89,90]. Studies on Eragrostideae, *Avena*, *Gentiana* section *Cruciata*, and *Pterocarpus* revealed that the variation of SC region in the plastome is greater than that of the IR region [30,40,56,67,72], and the same conclusion was obtained in this study.

Some high mutation regions in plastome sequences can be used as molecular markers for species identification and phylogenetic relationship analysis [91]. The chloroplast genes *rbc*L, *trn*H, *psb*A, and *mat*K have been considered as core plant barcodes for species identification in previous studies, but their resolution at a species level was usually limited [92]. Among the four core markers, only *mat*K was detected as being highly variable in Aristidoideae. The *ndh*F was screened as divergent marker in Aristidoideae, which has also been used as a maker in *Stipa* [93]. The identified makers *rps*3*2*, *mat*K, *ndh*A, *rpl*32-trnL-UA*G*, *ndh*F-*rpl*32, *trn*Y-GUA*-trn*D-GUC, and *ccs*A*-ndh*D in Aristidoideae also showed high variability in the study of *Avena* plastome [30]. Six reported potential markers in Eragrostideae, *ndh*F, *mat*K, *ndh*F*-rpl*32, *rpl*32*-trn*L-UAG, *trn*G-GCC*-trn*fM-CAU, and *ccs*A-ndhD, were also found to be highly variable in Aristidoideae [56]. Studies in different Poaceae taxa showed that there are indeed some shared potential markers in Poaceae. The 12 highly variable loci identified in this study will be potential markers for population genetics or phylogenetic studies in Aristidoideae.

### 4.3. Phylogenetic Relationships of Aristidoideae

In the current study, Aristidoideae is resolved as being a sister to Panicoideae, based on the alignment of 22 complete plastomes from the PACMAD clade. The position of Aristidoideae in the PACMAD clade has been controversial for a long time [3,4,12,94,95]. The crux of the dispute lies in whether the subfamily Panicoideae or Aristidoideae is the basal group of PACMAD clade, namely the ‘panicoid-sister hypothesis’ or the ‘aristidoid-sister hypothesis’ [1,11,12,96]. For example, within PACMAD, the ‘panicoid-sister hypothesis’ or the ‘aristidoid-sister hypothesis’ were supported based on plastome data matrices without or with alignment gaps, respectively [96].

The ML and BI phylogenetic trees of Aristidoideae were reconstructed using the plastome data of 19 species, including ten *Aristida* species, three *Stipagrostis* species, three *Sartidia* species, and three outgroup species. The monophyly of three genera in Aristidoideae is strongly supported (BS = 100, PP = 1). Phylogenetic analysis based on all plastome data matrices indicated that *Sartidia* is a sister to *Stipagrostis* (BS = 100, PP = 1) and then a sister to *Aristida* (BS = 100, PP = 1). However, the phylogenetic tree, based on six chloroplast gene fragments and *the* nuclear *ppc* multigene family, support *Aristida* and *Stipagrostis* as having formed a sister group [7,13]. The study, which combined chloroplast *trn*L-F, *rpl*16, and nuclear ITS sequences, support *Aristida* to be a sister to the clade composed of *Stipagrostis* and *Sartidia*, but only one *Sartidia* species was included [10]. In addition, some detailed clues in this study may also help to explain the closely-related relationship between *Sartidia* and *Stipagrostis*. In terms of the plastome structure, (1) all *Aristida* species have 120 bp *ycf*1 gene residues, while in *Sartidia* and *Stipagrostis* species, the *ycf*1 gene was completely degraded; (2) the *ndh*H gene, located in IRa, was 1182 bp in *Aristida* species, while its length in *Sartidia* and *Stipagrostis* species was 1188 bp. In terms of carbon assimilation, although both *Aristida* (except *A. longifolia*) and *Stipagrostis* perform C_4_ photosynthesis, there are differences both anatomically and in the phosphoenolpyruvate carboxylase for photosynthesis between the two genera [7]. All Aristidoideae species contain two layers of vascular bundle sheath cells. Two layers of vascular bundle sheath are parenchyma cells in *Aristida*, while in *Sartidia* and *Stipagrostis* only the outer layer of the vascular bundle sheath are parenchyma cells [3,5]. The estimated split time of *Stipagrostis* and *Sartidia* is 11.04 Ma (Miocene). Drought may have promoted the divergence of *Stipagrostis* and *Sartidia* [97].

*Aristida* is a large genus with more than 300 species, and there have been few studies on its inter-species relationship. The ten *Aristida* species sampled in this study are divided into two clades. The clade comprising *A. rufescens*, *A. adscensionis*, *A. congesta*, *A. diffusa*, and *A. stipitata* showed the same topologies in all data sets, which was consistent with previous studies [10]. Another clade was composed of *A. ternipes*, *A. glaziovii*, *A. purpurea*, *A. behriana*, and *A. pruinosa*. Our results indicated that plastome data can be used as potential super-barcode to reconstruct the interspecies relationships of *Aristida*. Within *Stipagrostis*, *St. pennata* was a sister to the clade comprising *St. hirtigluma* and *St. uniplumis* for all three datasets, which was also supported by the study based on chloroplast *trn*L-F, *rpl*16 and nuclear ITS [10]. In addition, two species, *Stipagrostis grandiglumis* and *Stipagrostis pennata*, originally belonging to *Aristida* in “Flora Reipublicae Popularis Sinicae”, have been classified into *Stipagrostis* in “Flora of China” [2,98]. In this study, only *Stipagrostis pennata* was obtained. The most notable morphological difference distinguishing *Stipagrostis pennata* from *Aristida* were the feathery hairs on the awns and the dense sandy sheath on the fibrous roots. Finally, the morphological features and all molecular evidence supported *Stipagrostis pennata* to be subsumed into *Stipagrostis*. For *Sartidia*, the interspecies relationship was not well resolved, which was identical to previous studies [9]. It was indicated that we need more data to illuminate the phylogenetic relationships among *Sartidia* species.

### 4.4. Evolutionary Implication of Aristidoideae

The diversification time of *Aristida* was inferred to be within the Miocene period [10]. The expansion of the savanna in the Miocene period may be related to the prosperity of *Aristida* [99,100,101]. *Stipagrostis* and *Sartidia* were split at 11.04 Ma (Miocene), which may be promoted by the drought event recorded in this period [97].

It is now generally accepted that, while there are only three genera in Aristidoideae, the differences in species diversity among the three genera are significant. There are more than 300 species in *Aristida*, more than 50 species in *Stipagrostis*, and only 6 species in *Sartidia*. *A*. *longifolia*, with C_3_ photosynthesis, is the first diverged taxon from *Aristida.* The distribution of *A. longifolia* is limited to the tropical area of central and southern South America. Both *Aristida* (except *A. longifolia*) and *Stipagrostis,* which have a high species richness, have C_4_ photosynthetic pathways. C_4_ photosynthesis is a group of complex traits that can increase photosynthetic efficiency under drought, high temperature, and low CO_2_ conditions. C_4_ photosynthesis may promote lineage species diversification by reducing extinction rates, increasing speciation rates, or combining the two [102], and have been proposed to be related to the high species diversity of grasses [7]. The distribution of these three genera is associated with the species numbers in each genus. *Aristida* is widely distributed in tropical and subtropical regions, indicating that *Aristida* can adapt to various habitats. *Stipagrostis*, distributed from Africa to Central and West Asia, is a kind of grass that can truly adapt to the desert environment. The seeds of *Stipagrostis* species have evolved some characteristics, such as a feathery pilose on the awn, to promote its wind spread in the desert [103]. In addition, polyploidization is a common phenomenon in plants, which can induce species diversity, promote speciation, and provide new genetic materials for plant evolution [104,105]. The ploidies of *Aristida* are varied, with diploid, triploid, and tetraploid structures, *Stipagrostis* are reported to have diploid and tetraploid, whereas *Sartidia* has only diploid [10]. The basic chromosome number of all three genera is 11 [3]. The occurrence of polyploidy and the variation of ploidy in *Aristida* and *Stipagrostis* may lead to species diversification in each genus.

## 5. Conclusions

In this study, the complete plastomes of *Aristida adscensionis* and *Stipagrostis pennata* were sequenced and assembled for the first time. Comparison of all 16 Aristidoideae plastomes found that they were highly conserved in genome size, gene number, structure, and IR boundary. A total of 12 highly variable regions were identified, which could be used as potential markers for phylogenetics, population genetics, and biogeography of Aristidoideae. In the present study, all phylogenetic trees strongly support the monophyly of Aristidoideae and three genera, and the clade of Aristidoideae and Panicoideae was a sister to other subfamilies in the PACMAD clade. Within Aristidoideae, *Aristida* is a sister to the clade composed of *Stipagrostis* and *Sartidia*. The phylogenetic relationships among sampled *Aristida* were well resolved. However, the interspecies relationships of *Sartidia* were still ambiguous, which indicated that nuclear data are needed for resolving the short internal branches. The divergence between C_4_ *Stipagrostis* and C_3_ *Sartidia* was estimated at 11.04 Ma which may be associated with the drought event in the Miocene period. The difference in species numbers of these three genera may be related to their difference in carbon fixation patterns, geographical distributions, and ploidy. In general, the plastome data used in this study provided insights into the phylogeny and evolution of the subfamily Aristidoideae.

## Figures and Tables

**Table 1:** The detailed information of the samples used in the present study.

Species	Accession Number	Species	Accession Number
*Amphipogon turbinatus*	NC_035521	*Cortaderia selloana*	NC_036681
*Aristida adscensionis*	MZ373986	*Eleusine coracana*	MW262987
*Aristida behriana*	NC_046729	*Eriachne mucronata*	NC_035529
*Aristida congesta*	NC_046731	*Isachne distichophylla*	NC_025236
*Aristida diffusa*	NC_046732	*Merxmuellera tsaratananensis*	NC_036122
*Aristida glaziovii*	NC_046413	*Oryza sativa*	NC_031333
*Aristida pruinosa*	NC_042836	*Sartidia dewinteri*	NC_027147
*Aristida purpurea*	NC_025228	*Sartidia isaloensis*	NC_036117
*Aristida rufescens*	NC_036130	*Sartidia perrieri*	NC_027146
*Aristida stipitata*	NC_046730	*Setaria viridis*	NC_028075
*Aristida ternipes*	NC_037164	*Sorghum bicolor*	NC_008602
*Arundo plinii*	NC_034652	*Stipagrostis hirtigluma*	NC_036112
*Bambusa bambos*	NC_026957	*Stipagrostis pennata*	MZ373985
*Brachyelytrum aristosum*	NC_027470	*Stipagrostis uniplumis*	MF460973
*Centropodia glauca*	NC_029411	*Thysanolaena latifolia*	NC_025238
*Chloris virgata*	NC_032034	*Zea mays*	NC_001666

**Table 2:** Plastome characteristics of 16 Aristidoideae species.

Species	Genome Size (bp)	LSC (bp)	IR (bp)	SSC (bp)	GC Content (%)				Number of Genes			
					All	IR	LSC	SSC	Total	CDS	rRNAs	tRNAs
*Aristida adscensionis*	137,947	80,085	22,722	12,418	38.4	43.9	36.2	32.4	134	88	8	38
*Aristida behriana*	137,074	79,444	22,568	12,494	38.6	44	36.4	32.6	134	88	8	38
*Aristida congesta*	134,072	80,384	20,670	12,348	38.3	44.1	36.2	32.6	134	88	8	38
*Aristida diffusa*	137,542	79,692	22,708	12,434	38.4	43.9	36.2	32.5	134	88	8	38
*Aristida glaziovii*	132,603	80,193	19,963	12,484	38.4	44.3	36.3	32.6	134	88	8	38
*Aristida pruinosa*	137,353	79,421	22,722	12,488	38.5	44	36.4	32.6	134	88	8	38
*Aristida purpurea*	138,423	80,437	22,747	12,492	38.5	43.9	36.3	32.6	134	88	8	38
*Aristida rufescens*	137,659	79,718	22,719	12,503	38.5	43.9	36.4	32.6	134	88	8	38
*Aristida stipitata*	138,226	80,327	22,728	12,443	38.4	44	36.2	32.4	134	88	8	38
*Aristida ternipes*	136,231	80,283	21,724	12,500	38.5	44.1	36.4	32.5	134	88	8	38
*Sartidia dewinteri*	138,074	80,116	22,725	12,508	38.4	44	36.2	32.3	132	86	8	38
*Sartidia isaloensis*	138,607	80,567	22,724	12,592	38.4	43.9	36.2	32.3	132	86	8	38
*Sartidia perrieri*	138,471	80,508	22,730	12,503	38.4	44	36.2	32.3	132	86	8	38
*Stipagrostis hirtigluma*	138,026	80,137	22,748	12,393	38.5	43.9	36.3	32.9	132	86	8	38
*Stipagrostis pennata*	138,725	80,927	22,679	12,440	38.4	44	36.4	32.8	132	86	8	38
*Stipagrostis uniplumis*	138,019	80,031	22,797	12,394	38.5	43.9	36.4	32.8	132	86	8	38

**Table 3:** Correlation analysis between cpSSRs and plastome size and CG content in 16 Aristidoideae species.

Term	Plastome Size	GC Content	Total SSRs	P1%	P2%	P3%	P4%	P5%	P6%
Plastome Size	1								
GC content	0.2473	1							
Total SSRs	−0.4087	0.1868	1						
P1%	−0.589 *	−0.0325	0.766 **	1					
P2%	0.661 **	−0.1420	−0.3807	−0.590 *	1				
P3%	0.0761	0.565 *	−0.0616	−0.0010	−0.1244	1			
P4%	0.3473	0.0234	−0.677 **	−0.845 **	0.2253	−0.2124	1		
P5%	0.1996	−0.1588	−0.2326	−0.2800	0.2742	0.1881	−0.1428	1	
P6%	0.0992	−0.1604	0.2575	−0.0327	0.3447	−0.3606	−0.0262	−0.16742	1

Note: * *p* < 0.05; ** *p* < 0.01.

**Table 4:** The codon usage of 16 Aristidoideae plastomes.

Species	CC	ENC	GC	T3s	C3s	A3s	G3s
*Aristida adscensionis*	16,986	49.51	0.389	0.4643	0.1712	0.4263	0.1739
*Aristida behriana*	17,016	49.75	0.39	0.4631	0.1728	0.4237	0.177
*Aristida congesta*	17,004	49.63	0.39	0.4637	0.1719	0.425	0.175
*Aristida diffusa*	17,004	49.65	0.39	0.4638	0.1721	0.4242	0.1758
*Aristida glaziovii*	17,005	49.79	0.391	0.4627	0.1726	0.4235	0.1772
*Aristida pruinosa*	17,023	49.72	0.39	0.4631	0.1722	0.4245	0.1767
*Aristida purpurea*	17,003	49.89	0.391	0.4615	0.174	0.4233	0.1774
*Aristida rufescens*	17,066	49.82	0.391	0.4622	0.1727	0.4234	0.1777
*Aristida stipitata*	17,011	49.66	0.39	0.4639	0.172	0.4245	0.1758
*Aristida ternipes*	17,001	49.87	0.391	0.4617	0.1738	0.4226	0.1779
*Sartidia dewinteri*	17,072	49.62	0.39	0.4636	0.1723	0.4257	0.1744
*Sartidia isaloensis*	17,034	49.55	0.39	0.4641	0.1718	0.4257	0.1739
*Sartidia perrieri*	17,034	49.57	0.39	0.464	0.1719	0.4251	0.1744
*Stipagrostis hirtigluma*	17,101	49.74	0.391	0.4616	0.1756	0.4243	0.1744
*Stipagrostis pennata*	17,055	49.75	0.391	0.4613	0.1753	0.4249	0.174
*Stipagrostis uniplumis*	17,101	49.73	0.391	0.4618	0.1755	0.4242	0.1745

Note: CC, Codons count; ENC, Effective number of codons; GC, GC content at coding positions; N3s, the third base of codons.

## Data Availability

All the sequencing data generated in this study has been deposited in GenBank with accession numbers MZ373985 and MZ373986 (https://www.ncbi.nlm.nih.gov/genbank/ (accessed on 27 September 2021)).

## Supplementary Materials

The following are available online at https://www.mdpi.com/article/10.3390/biology11010063/s1, Figure S1: The ML tree of PACMAD based on complete plastomes. Figure S2: The ML tree of 16 Aristidoideae species based on CDS. Figure S3: The BI tree of 16 Aristidoideae species based on CDS. Figure S4: The ML tree of 16 Aristidoideae species based on IGS. Figure S5: The BI tree of 16 Aristidoideae species based on IGS. Table S1: SSRs types and numbers of 16 Aristidoideae plastomes. Table S2: SSRs location in 16 Aristidoideae plastomes. Table S3: The information of SSRs parsimony information locus. Table S4: Codon usage analysis of plastome protein coding genes in 16 Aristidoideae species.
