# Supplementary figures and images for: Comparative and Phylogenetic Analysis of Complete Plastomes among Aristidoideae Species (Poaceae)
Source: Biology (Basel). 2022 Jan 2;11(1):63. doi: 10.3390/biology11010063 (PMC8773369; doi:10.3390/biology11010063)

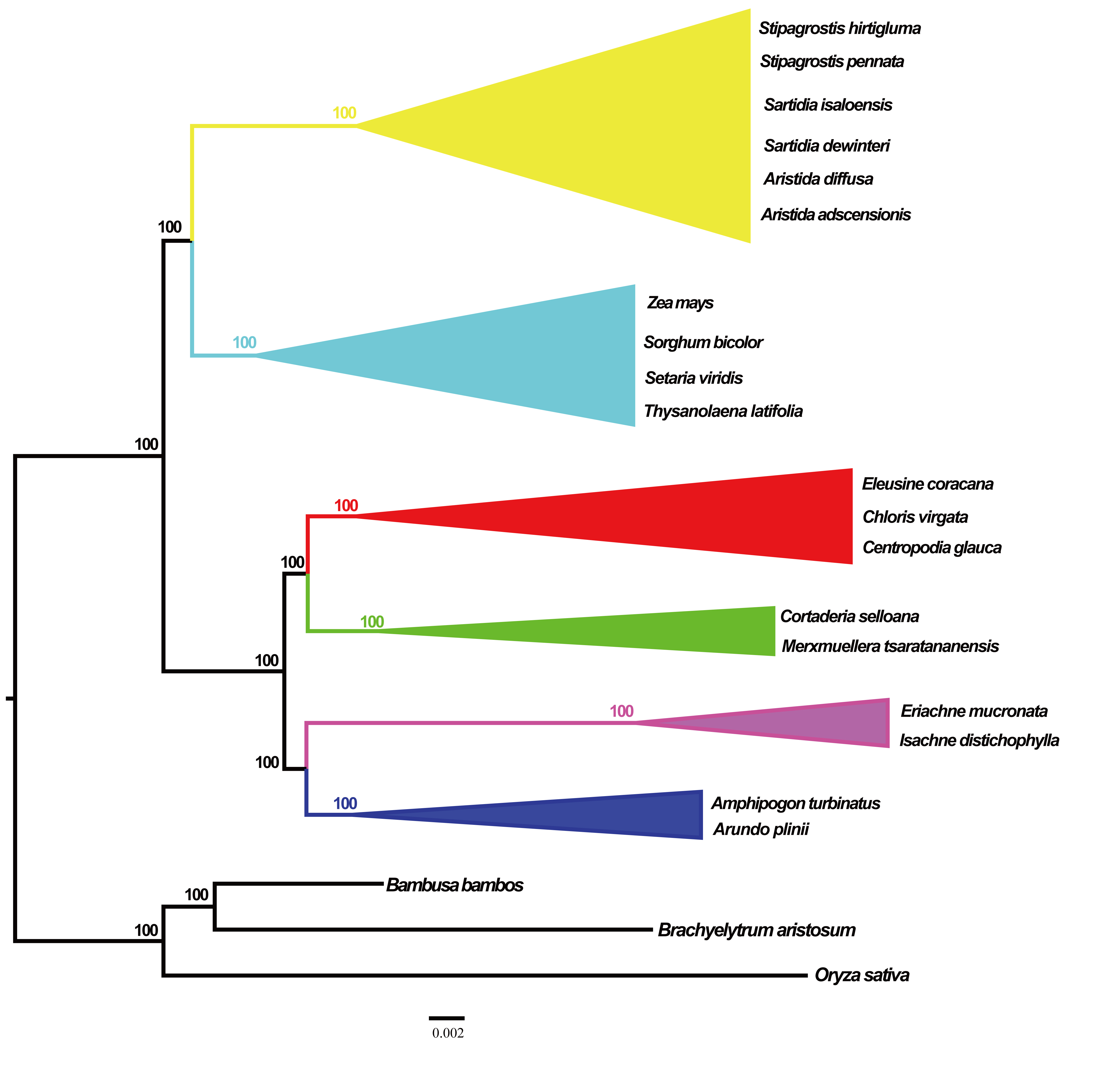

Supplement: Supplementary file 1 [file biology-11-00063-s001.zip › Figure S1.jpg]

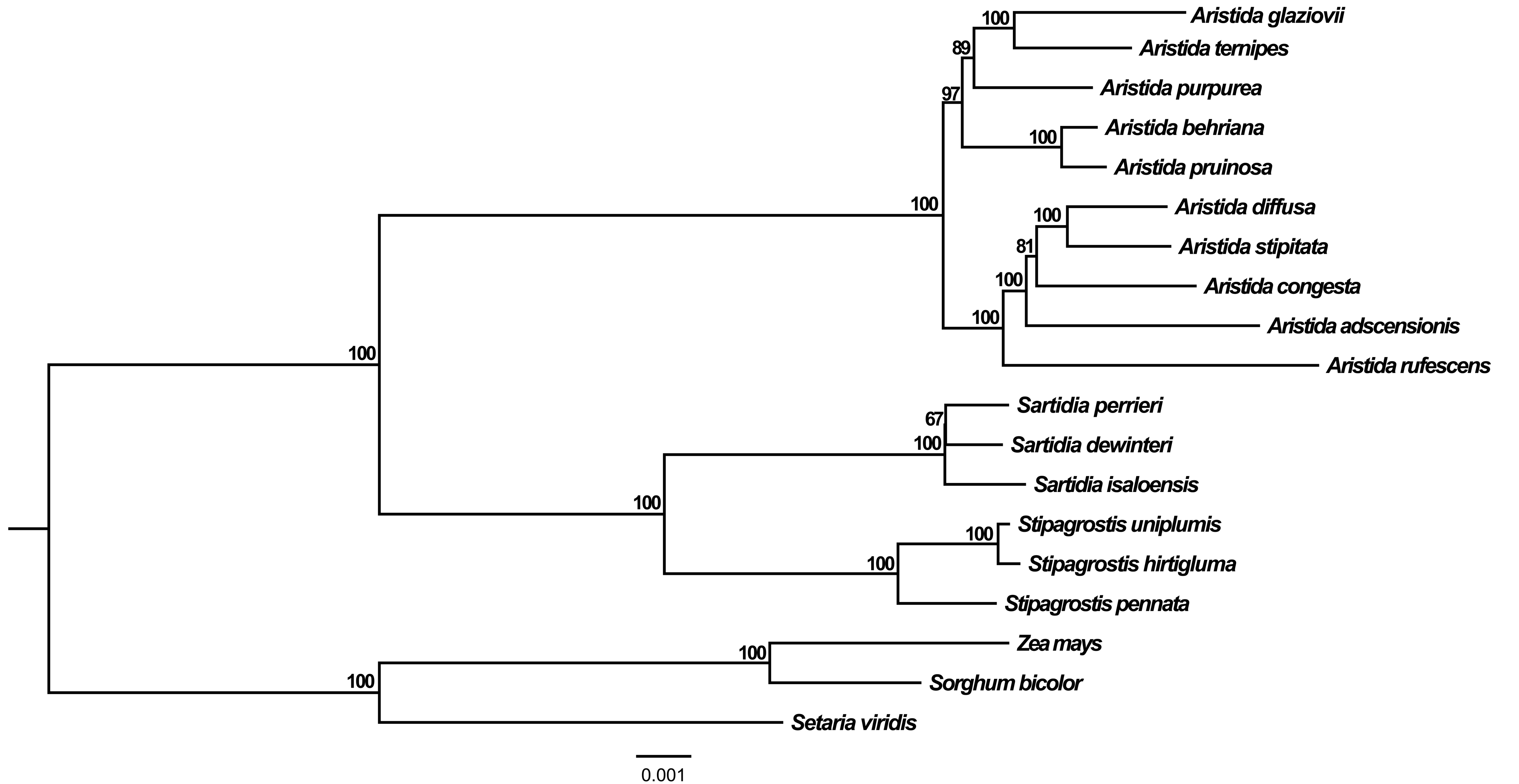

Supplement: Supplementary file 1 [file biology-11-00063-s001.zip › Figure S2.jpg]

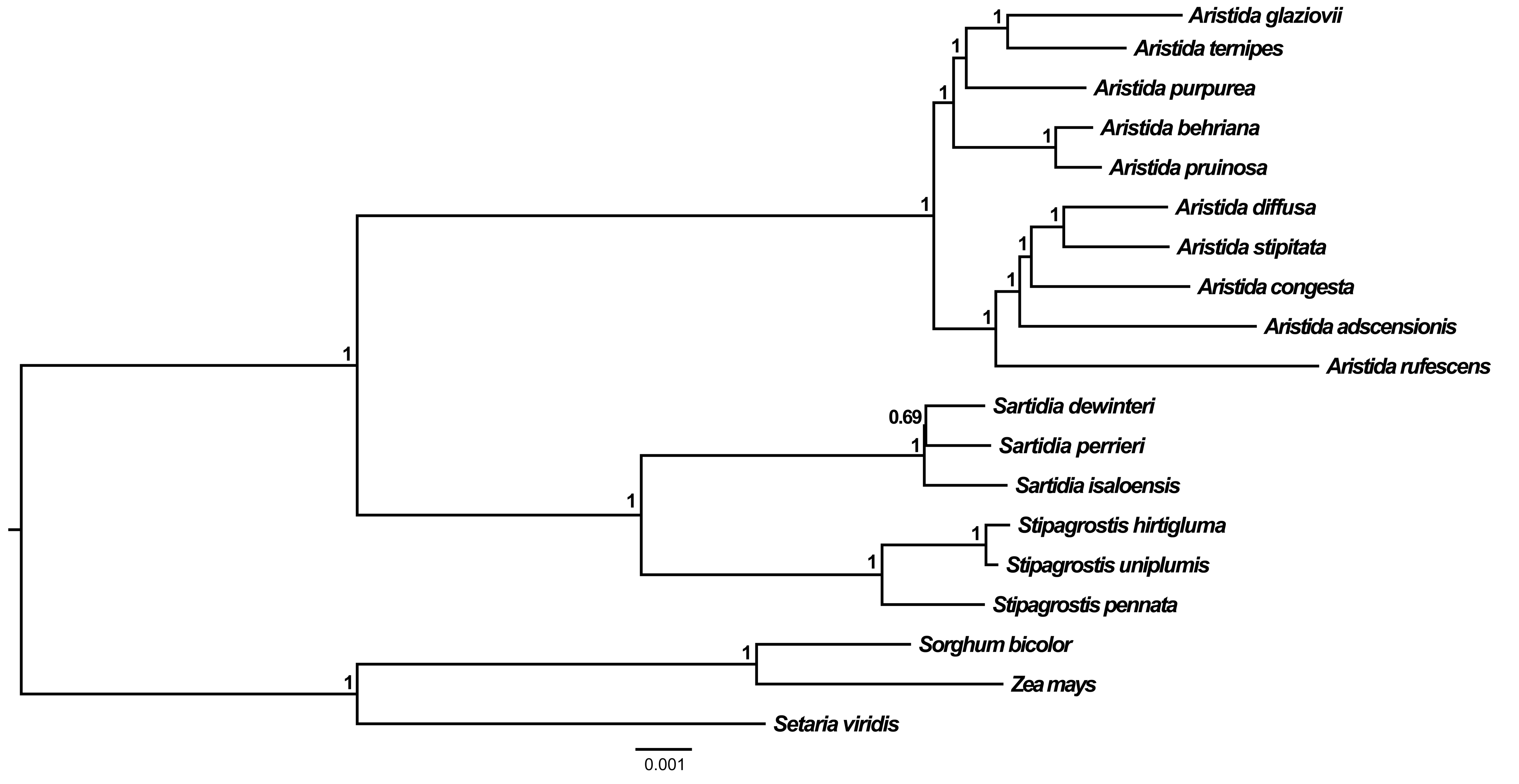

Supplement: Supplementary file 1 [file biology-11-00063-s001.zip › Figure S3.jpg]

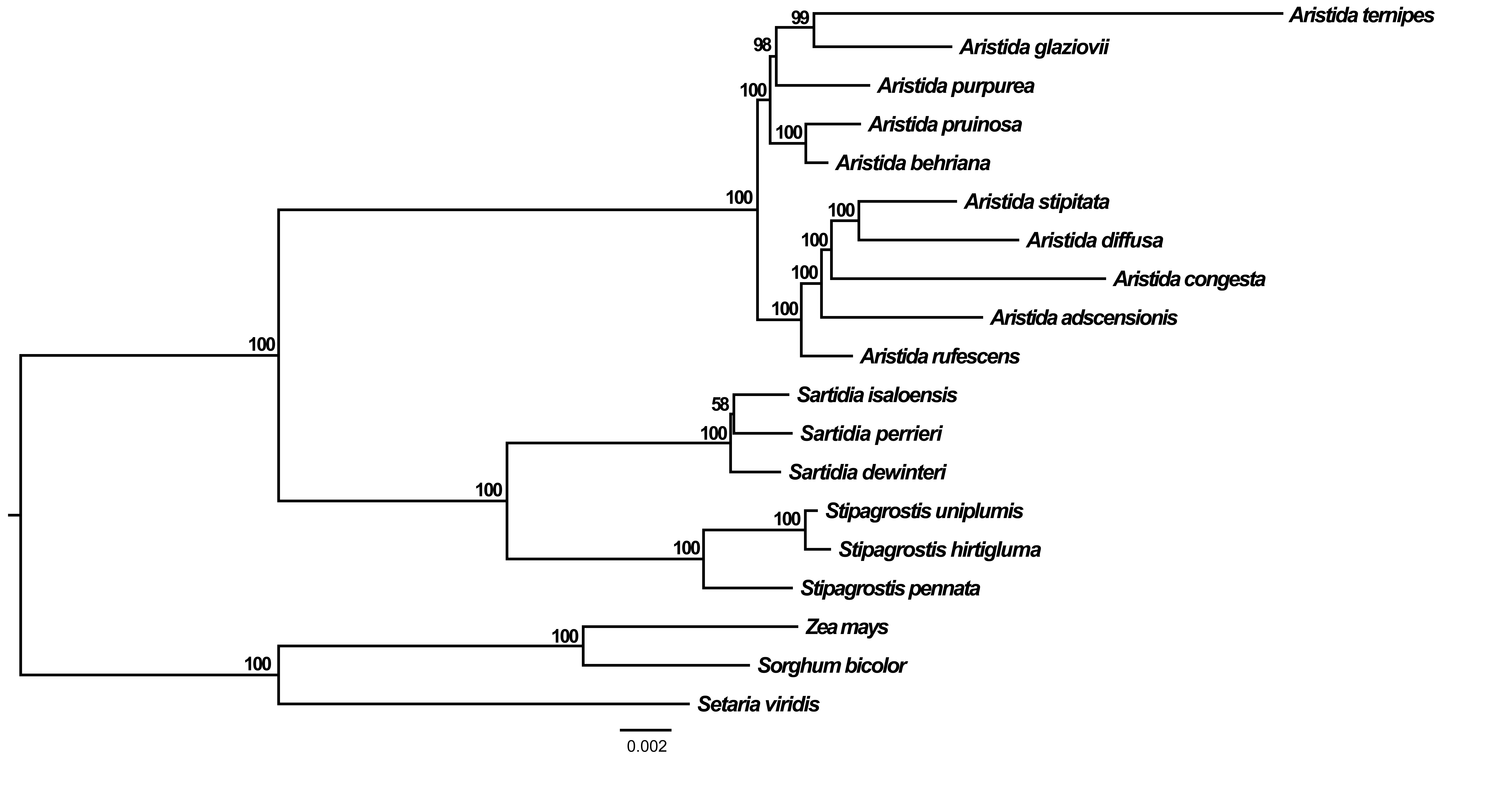

Supplement: Supplementary file 1 [file biology-11-00063-s001.zip › Figure S4.jpg]

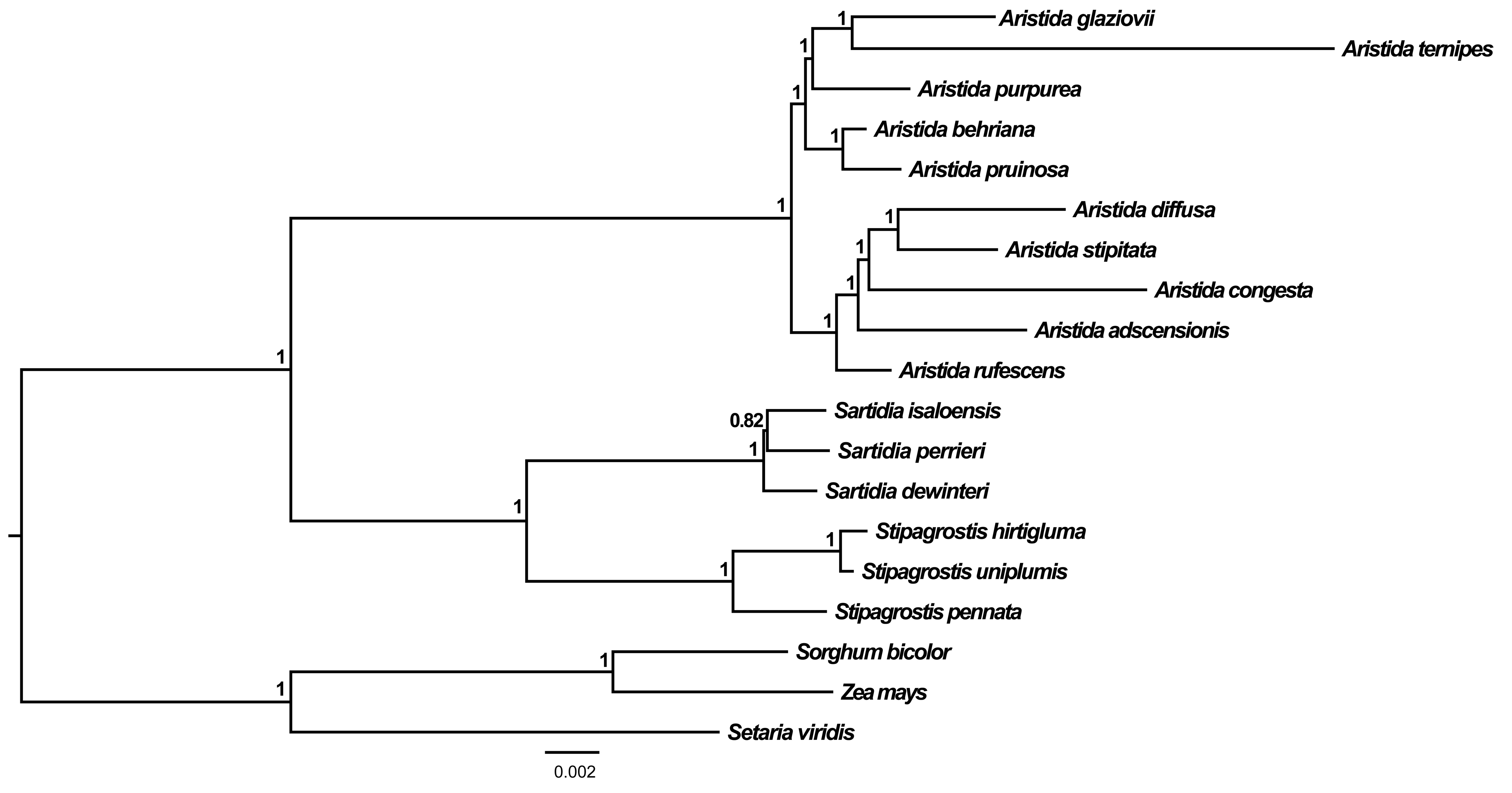

Supplement: Supplementary file 1 [file biology-11-00063-s001.zip › Figure S5.jpg]
